# Acute Myelomonocytic Leukemia Presenting as Perianal Pain

**DOI:** 10.1155/2018/2151492

**Published:** 2018-11-27

**Authors:** Syma Iqbal, James Walcott, Stephen Chan

**Affiliations:** ^1^Western Health, Footscray, Victoria 3030, Australia; ^2^Western Health, Sunshine, Victoria 3020, Australia; ^3^Dorevitch Pathology, Gordon Street, Footscray Hospital, Victoria 3011, Australia

## Abstract

Extramedullary involvement of the gastrointestinal tract (GIT) is a rare entity as most patients present with lymphoreticular organ involvement. Its detection and diagnosis can be extremely challenging, as these patients would present with unusual clinical symptoms. We diagnosed and managed a patient with leukemic infiltration of GIT who presented with perianal pain. Prompt use of MRI played an important role in detecting underlying pathology, and effective tissue sampling confirmed the diagnosis. This resulted in overall successful management of the patient.

## 1. Introduction

Extramedullary involvement of the gastrointestinal tract (GIT) with leukemia is relatively rare and not easily detectable [1]. However, it must be considered in patients with acute or chronic leukemia who present with unusual GIT symptoms if the etiology is not immediately evident [2].

## 2. Case Report

We present a case of a 70-year-old male, who presented with two-week history of perianal pain accompanied by urgency to defecate and anorexia. He was passing small stools with severe pain, sweating, and burning sensation. There was no history of abdominal pain, constipation, rectal bleeding, fever, and diarrhea or weight loss. Past history included coronary artery bypass graft and hiatus hernia repair. He had worked at a cattle farm and was a nonsmoker.

At presentation, he was haemodynamically stable with normal systemic examination. The perineum looked unremarkable with no blood or mucous. Digital examination was aborted due to pain. His hemoglobin was normal with raised inflammatory markers. Liver function tests were mildly elevated. A provisional diagnosis of deep perianal abscess was made.

**Figure 1:**
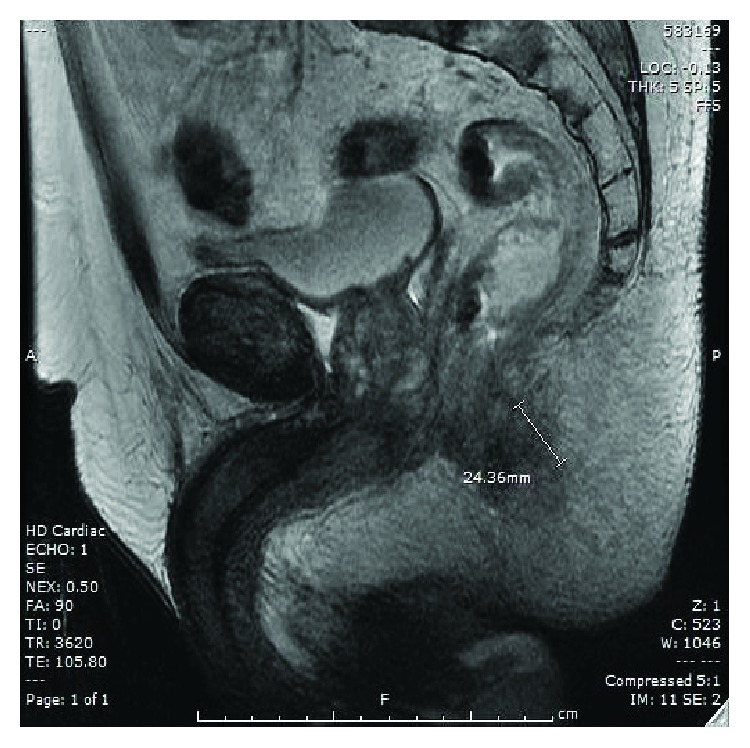
Lateral view of pelvic MRI showing inflammatory changes involving mucosa of the anus, rectum, and internal sphincter.

Following this, magnetic resonance imaging (MRI) was done which demonstrated inflammatory changes involving mucosa of the anus, rectum, and internal sphincter with no definite fistulous tract or collection (see Figure 1). His blood film showed marked monocytosis, promonocytes, and some circulating blasts suggesting acute myelomonocytic leukemia (AMML) or chronic myelomonocytic leukemia (CMML) in transformation.

**Figure 2:**
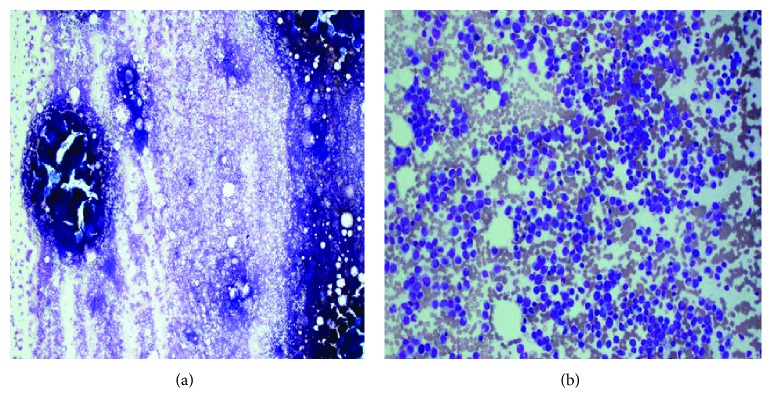
Hypercellular bone marrow aspirate showing marked excess of blasts.

**Figure 3:**
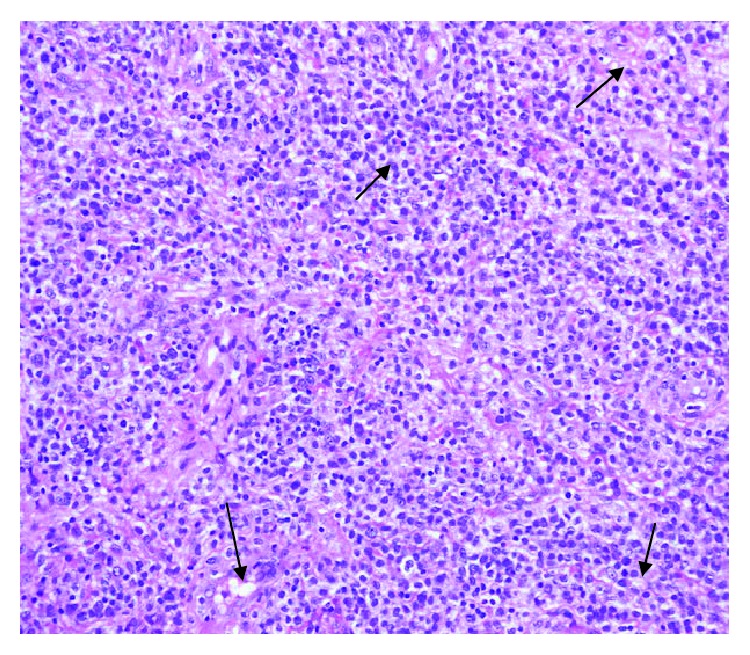
Anal tissue: H&E stain low power showing the tumor consisting of blasts with scant cytoplasm and round-oval nuclei with fine dispersed chromatin and distinct nucleoli. Mitotic figures are numerous.

**Figure 4:**
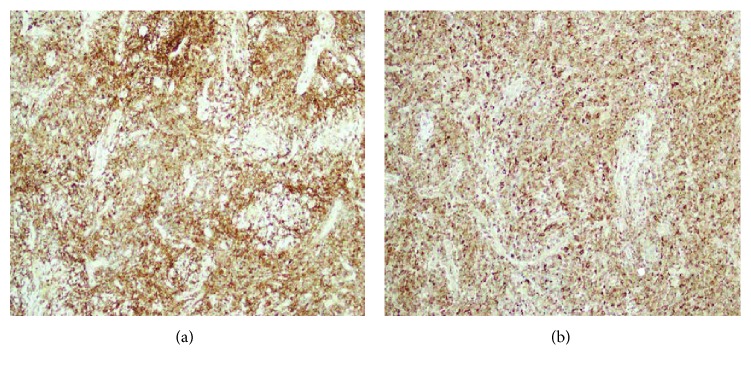
Neoplastic cells strongly express MPO.

Following admission, he became febrile; hence, full septic screen was done before starting intravenous antibiotics. An examination under anesthesia in theatre showed a generally inflamed, edematous rectum without contact bleeding. There was no mass, fistula, or abscess identified. A rectal polyp found at 5 o'clock position was excised and sent for histology. Several biopsies of the inflamed mucosa were also sent for histological analysis (Figure 2). Histology of the polyp revealed dense stromal infiltrate of atypical cells with granular eosinophilic cytoplasm, medium-large nuclei with irregular nuclear membrane, and prominent nucleoli, morphology, and immunohistochemical profile consistent with mucosal involvement by myelomonocytic leukemia. Immunoperoxidase stains showed CD4+, CD68+, CD45+, and MPO+ (see Figures 3 and 4).

The hematologist performed a trephine bone marrow biopsy that reported markedly hypercellular bone marrow aspirate showing excess blasts consistent with the diagnosis of acute leukemia, morphologically AMML. Flow cytometry showed an increased population detected with the phenotypes CD13+, CD33+, CD34−, CD65+, HLADr+, and CD117+ and aberrant expression of CD7+. His cytogenetic studies showed no molecular evidence of a translocation involving the KMT2A (MLL) gene at 11q23. No molecular/next generation sequencing was performed because of involvement of high cost of the test. Diagnosis of acute myeloid leukemia was hence made. Histology and bone marrow trephine biopsy results showed French American British (FAB) classification [3] as AMML-M4 and as World Health Organization (WHO) classification 2008 [4]. The patient was then transferred to a cancer hospital where he was offered intensive chemotherapy for AMML. He went into remission following chemotherapy, and his GIT symptoms settled subsequently.

## 3. Discussion

Extramedullary involvement of GIT with leukemia is rare as most patients are more likely to present with involvement of lymphoreticular organs and sanctuary sites such as the brain, testes, and ovaries. When it does occur, leukemic involvement of GIT has been described in locations from the mouth to the anus, in both solid and hollow organs.

Iwamoto et al. [5] comment that among leukemia, monocytic leukemia is more likely to present with extramedullary disease. They report that involvement of the colon can present with abdominal pain, bleeding, diarrhea, or obstruction. Its manifestations encompass polypoid lesions, rectal watermelon vasculopathy, and colitis.

The reported autopsy incidence of gastrointestinal involvement by leukemia ranges from 5.7%–13% [6]. The incidence rate is 1–2/100,000/year [7] with median age of the onset is 65–70 years [8–10]. Specific etiological factors of the disease are unclear but may be associated with the exposure to ionizing radiation [11], occupational and environmental carcinogens or toxins. 20–30% of the patients are asymptomatic with the disease suspected from routine blood tests [12–15]. Among symptomatic patients, 20% have been reported to have weight loss, 15% excessive sweating, and 15% abdominal fullness [16].

Cornes and colleagues [17] concluded in their study that most commonly involved extramedullary sites were the stomach, ileum, and proximal colon. Macroscopically, it can present as necrosis, hemorrhage, ulceration, or polypoid lesions.

Leukemic involvement of the GIT must be confirmed histologically. Michiko et al. highlight the importance of tissue sampling in their paper and stress that biopsies should be taken on all abnormal-appearing and sometimes normal-appearing tissues for histologic confirmation of suspected pathology.

The challenge with this patient was getting an initial diagnosis, as he had no prior diagnosis of leukemia. The course of disease experienced by this patient suggested that GIT involvement with leukemia might parallel leukemic disease activity, as acceleration of the patient's WBC coincided with a worsening of his symptoms. Appropriate treatment of the underlying leukemia addressed his symptoms.

## 4. Conclusion

Leukemic infiltration of the colon although rare should be considered whenever there is suspicion of AML on the blood film in a patient presenting with GIT symptoms. Appropriate examination and histological diagnosis by tissue sampling should always be considered in the management of patients presenting with perianal pain where the diagnosis is unclear.
